# Adherence to treatment and related factors among patients with chronic conditions in primary care: a cross-sectional study

**DOI:** 10.1186/s12875-019-1019-3

**Published:** 2019-09-14

**Authors:** Cesar I. Fernandez-Lazaro, Juan M. García-González, David P. Adams, Diego Fernandez-Lazaro, Juan Mielgo-Ayuso, Alberto Caballero-Garcia, Francisca Moreno Racionero, Alfredo Córdova, Jose A. Miron-Canelo

**Affiliations:** 1 Department of Biomedical and Diagnostic Sciences, School of Medicine, University of Salamanca, Calle Alfonso X el Sabio s/n, 37007 Salamanca, Spain; 2 Department of Preventive Medicine and Public Health, School of Medicine, IDISNA, University of Navarra, Pamplona, Spain; 3 Department of Sociology, Pablo de Olavide University, Seville, Spain; 4 Dual Enrollment Program, Point University-Savannah Campus, Savannah, GA USA; 5 Department of Cell Biology, Histology and Pharmacology, University of Valladolid, Soria, Spain; 6 Department of Biochemistry, Molecular Biology and Physiology, Faculty of Physical Therapy, University of Valladolid, Soria, Spain; 7 Department of Anatomy and Radiology, University of Valladolid, Soria, Spain; 8 Castile and Leon Healthcare Services, Valladolid, Spain

**Keywords:** Treatment adherence, Medication adherence, Patient adherence, Patient education, Chronic conditions, Multidimensional factors, WHO multidimensional framework, Primary care

## Abstract

**Background:**

Adherence to treatment, a public health issue, is of particular importance in chronic disease therapies. Primary care practices offer ideal venues for the effective care and management of these conditions. The aim of this study is to assess adherence to treatment and related-factors among patients with chronic conditions in primary care settings.

**Methods:**

A cross-sectional study was conducted among 299 adult patients with ≥1 chronic condition(s) and prescribed medication in primary healthcare centers of Spain. The Morisky-Green-Levine questionnaire was used to assess medication adherence via face-to-face interviews. Crude and adjusted multivariable logistic regression models were used to analyze factors associated with adherence using the Multidimensional Model proposed by the World Health Organization — social and economic, healthcare team and system-related, condition-related, therapy-related, and patient-related factors.

**Results:**

The proportion of adherent patients to treatment was 55.5%. Older age (adjusted odds ratio 1.31 per 10-year increment, 95% CI 1.01–1.70), lower number of pharmacies used for medication refills (0.65, 95% CI 0.47–0.90), having received complete treatment information (3.89, 95% CI 2.09–7.21), having adequate knowledge about medication regimen (4.17, 95% CI 2.23–7.80), and self-perception of a good quality of life (2.17, 95% CI 1.18–4.02) were independent factors associated with adherence.

**Conclusions:**

Adherence to treatment for chronic conditions remained low in primary care. Optimal achievement of appropriate levels of adherence through tailored multifaceted interventions will require attention to the multidimensional factors found in this study, particularly those related to patients’ education and their information needs.

**Electronic supplementary material:**

The online version of this article (10.1186/s12875-019-1019-3) contains supplementary material, which is available to authorized users.

## Background

The increase in life expectancy and the aging of the world population have been paralleled by an alarming growth in the global burden of chronic conditions [1]. Chronic diseases are generally considered physical or mental conditions that last more than a year and require ongoing care. They compromise the individuals’ physical and social function, the health-related quality of life, and the economic sustainability of healthcare systems [2, 3]. Their global prevalence has reached such unprecedented levels in many populations that chronic diseases currently represent a public health concern. Roughly a quarter of the European population suffers from at least one chronic condition, and an estimated 50 million people suffer from multimorbidity [4], the co-occurrence of two or more chronic diseases [5]. In the United States, chronic conditions affect 60% of American adults, and four in ten suffer from multimorbidity [6].

For people with chronic diseases, management of their conditions is fundamental to minimize their impact, improve health outcomes, prevent further disability, and reduce healthcare costs [7, 8]. Adherence to treatment, the extent to which patients are able to follow the agreed recommendations for prescribed treatments with healthcare provider, is a key component of chronic disease management. Only half of patients with chronic conditions, however, take their medications as prescribed, making medication adherence improvement a priority of the public health agenda [9]. According to the World Health Organization (WHO), a series of factors, rather than a single one, determine patients’ ability to follow treatment recommendations correctly. These factors interact and potentiate each other’s influence in a framework determined by five dimensions — the social and economic, health-care team and system-related, condition-related, therapy-related, and patient-related [9].

Several attempts have been made in recent years to determine the most influential factors of adherence. Most research has focused on a single-dimension, usually patient-related factors [10] and have not taken into account the WHO framework. Other studies have focused on a single-disease, such as diabetes [11], coronary heart disease [12], and asthma [13], or on a particular drug therapy [14], an approach which limits the utility of the findings to the condition under study. To identify facilitators of adherence among chronic-disease patients, it is necessary to consider more than a single chronic condition and account for interaction of factors in a more multi-dimensional approach.

Spain has one of the highest prevalence of multimorbidity [4, 15, 16] in Europe. Present demographic trends suggest its population will have the longest life expectancy in the world by 2040 [17]. Nonetheless, no studies have used a multi-dimensional approach to evaluate factors associated with medication adherence in primary care settings, venues that provide most of the care and management of chronic conditions [18–20]. Moreover, some authors have also emphasized the need to develop studies in the context of primary care to better assess medication adherence, as these places offer more accurate results and minimize selection bias [21]. Thus we aim to use the WHO conceptual framework to evaluate adherence and related factors among chronic-disease patients in these settings. Their assessments can guide interventions that will reduce healthcare costs and improve patients’ health-related quality of life.

## Methods

### Study design and settings

A cross-sectional study was conducted in two primary healthcare centers between August 2016 and March 2017 in Soria, an urban city of 39,000 inhabitants located in the autonomous community of Castile and Leon, Spain. The Spanish National Health System (SNHS) provides universal health coverage to all Spanish citizens and foreign nationals and has two levels of care: Primary Health Care and Specialist Care.

Primary Health Care is distributed in health areas that provide basic health care services through one or more healthcare centers. All centers operate strive to follow the same principles to maximize healthcare accessibility and equity within the country. Healthcare centers in Spain offer services free of charge at the point of delivery such as consultations, health education, laboratory tests, physical therapy, and radiographic exams [22]. Currently, the facilities of the study are the main healthcare centers of the Health Area of Soria and provide healthcare services to the urban population of the city, one of the 52 Spanish provincial capitals.

### Population and sampling

The sample population of the study was calculated based on the number of patients that attended the clinics and an estimated prevalence of chronic conditions among primary care population of 70% [16]. We accepted an expected proportion of the participants not adhering to prescribed medications of 75% [23, 24], keeping a 95% confidence level, a 5% tolerated error level, and a design effect of 1. Taking into account a possible refusal rate of 20%, the final sample size was calculated to be 344 patients.

The investigators screened potential candidates for participation at each center. After candidates’ screening, research assistants randomly approached potential participants presenting at the centers for follow-up consultations, confirmed their eligibility, and invited them to participate in the study. Patients aged 18 years or older who suffered from one or more chronic condition(s) and had been prescribed medication for more than a year of treatment were considered eligible for inclusion in the study. Individuals with cognitive impairment or mental conditions that prevented them from responding appropriately, and those who declined to participate, were excluded from the study. A total of 23 health problems were defined as chronic conditions in the study (Additional file 1: Table S1). These conditions were selected because they are typically treated with prescribed medications and commonly occur in primary care settings.

### Data collection

A questionnaire, previously designed by an expert group in medication adherence, was used to obtain information about patients’ socio-demographic characteristics and factors related to adherence. Prior to its implementation, investigators piloted the survey on 30 subjects to ensure that it was easily understood, well-defined, and accurately addressed the goals of the study.

Data collection took place in a clinic consultation room after participants’ appointment with their healthcare providers. Face-to-face, semi-structured interviews, were conducted by a pharmacist with extensive experience in adherence surveys. Each interviewer-patient session lasted between 20 and 30 minutes. Participation in the study was voluntary and all the subjects received a detailed explanation about the goals, objectives, methods, and purpose of the study. Patients, whose responses were coded to protect their confidentiality, were also informed they could withdraw from the study at any time without penalty.

The Ethics Committee of the Hospital Complex of Soria approved this study. All participants provided written informed consent to take part in the study.

### Measurement of exposure and covariates

Self-reported adherence was determined using the Spanish validated version [25] of the four-item Morisky-Green-Levine Medication Assessment Questionnaire [26]. This method is simple, easy to implement, and has the ability to identify reasons underlying the medication adherence behavior. Furthermore, it has been widely used in numerous studies and clinical settings. The questionnaire consists of 4 yes/no questions: (i) Do you ever forget to take your medicine? (ii) Are you careless at times about taking your medicine? (iii) When you feel better, do you sometimes stop taking your medicine? and (iv) If you feel worse when you take the medicine, do you stop taking it? Each “*yes”* response received a score of “1”, and each “*no”* response received a score of “0”. Patients’ overall medication adherence was categorized as follows: ≥3 score = low adherence; 1–2 score = medium adherence; and 0 score = high adherence.

The following factors were considered for their possible association with medication adherence using the WHO multidimensional framework [9]:

- *Social and Economic Factors*

Gender, age, immigration status (born in Spain vs. immigrated to Spain), household income (tertiles), living status (living alone vs. living with someone), and highest level of education (primary school or lower, secondary school, and university or higher) were included as social economic factors.

- *Healthcare Team and System-Related Factors*

Information about patients’ frequency of follow-up care for chronic diseases (monthly or more frequently, quarterly, and biannually or less frequently) and the number of pharmacies used for medication refills were considered. Additionally, patient-provider communication, perceived quality of healthcare delivery, and level of treatment information received were evaluated on a 5-point Likert scale: from “1″ meaning *very poor* to “5″ meaning *very good.* Scores above “3″ were used to categorize respondents as *having good communication with provider, perceiving good quality of healthcare delivery*, and *receiving complete treatment information* for each item respectively.

- *Condition-Related Factors*

Data about condition-related factors included the number of chronic conditions, adjusted morbidity group (AMG) based on the Clinical Risk Group classification [27], and lifestyle behavior such as alcohol consumption, tobacco smoking use, and levels of physical activity. The AMG is a new multi-morbidity risk adjustment measure of disease severity, number of diseases, healthcare services utilization, and difficulties associated with access to resources. This measure has been adapted to the Spanish Health System and has been used by the Castile and Leon Healthcare Services since 2013 to manage patients with chronic conditions [28].

Claim-based diagnoses were used to assign subjects to a mutually exclusive, hierarchically ranked risk, group burden of comorbid diseases: AMG1 (single minor chronic condition), AMG2 (multimorbidity with stable chronic conditions), and AMG3 (complex multimorbidity with severe chronic conditions). Regarding lifestyle, participants self-reported their frequency of alcohol consumption (daily drinker, occasional drinker, and non-drinker), tobacco smoking (smoker, ex-smoker, and non-smoker), and physical activity during leisure time (active vs. non-active) according to the WHO recommendations [29].

- *Therapy-Related Factors*

The number of prescriptions, pills, and use of medication by injections (use vs. non-use injections), and by inhalers (use vs. non-use inhalers) included in the treatment of each participant were considered as therapy-related factors. Moreover, patients reported on a 5-point Likert scale the degree to which treatment interfered with their activities of daily living with “1” meaning *not any interference* to “5” meaning *very much interference.* Scores above “3” were used to categorize treatment respondents viewed as *interfering with activities of daily living*.

- *Patient-Related Factors*

Information collected about patient-related factors reflected in participants’ functional independency of activities of daily living evaluated using the Barthel Index: independent, slightly dependent, moderately dependent, severely dependent, and totally dependent [30]; the use of aids to remember medication-dosing schedules (no reminders, alarms/phones/pillboxes, and association of medication with daily routines); knowledge of medication regimen, i.e., specific amount, number, and frequency of doses (adequate vs. inadequate knowledge); and whether patients perceived overmedication in their treatment (perception vs. no-perception of overmedication). Individuals also reported their self-perceived quality of life on a 5-point Likert scale with “1″ meaning *very poor* to “5″ meaning *very good*. Scores above “3″ were used to categorize respondents as *having good quality of life.*

### Data management and statistical analysis

For statistical analyses participants were categorized according to their responses of the Morisky-Green-Levine questionnaire into an “adherent group” (questionnaire’s score = 0/high adherence) and a “poor-adherent group” (questionnaire’s score ≥ 1/medium and low adherence). Descriptive statistics included means and standard deviations (SD) for quantitative variables and percentages for categorical variables. We performed crude and adjusted multivariate logistic regression to evaluate factors associated with medication adherence. Covariates were included in the multivariate logistic regression model based on the crude association with adherence (*p*-value ≤ 0.05). These variables were age, immigration status, living status, number of pharmacies used for medication refills, treatment information received, number of chronic conditions, adjusted morbidity group, interference of therapy on daily life activities, medication dosing reminders, patients’ knowledge about medication regimen, and self-perceived quality of life. Sensitivity analyses were additionally performed to confirm the relationship between risk factors and adherence. No issues of multicollinearity were observed. These analyses were performed using Stata software, version 14.0 (StataCorp LP, College Station, TX) with a two-tailed level of statistical significance set at *p*-value ≤ 0.05.

## Results

### Sample characteristics

Among the 344 eligible patients randomly invited to participate in the study, 41 (11.9%) refused to participate and 4 (1.2%) withdrew during the interview process. The characteristics of the excluded subjects were similar to those of the overall group. The mean age of participants was 65.8 ± 13.7 years; most were male (51.5%) and born in Spain (83.9%). Nearly half had at least graduated from secondary school (41.1%). Participants had a mean number of 3.9 ± 2.2 prescriptions in their treatment and suffered an average of 2.9 ± 1.5 chronic conditions (Table 1). The most common chronic conditions among participants were circulatory system diseases (71.2%), followed by endocrine and metabolic disorders (53.2%), and mental and nervous system diseases (37.8%) (Additional file 1: Table S1).

**Table 1:** Social and Economic-, Healthcare Team and System-, and Condition-Related Factors of the Participants of the Study according to their Self-Reported Measure of Medication Adherence, *n* = 299

Factors	Total n (%)	Adherent n (%)	Poor Adherent n (%)	Bivariate Analyses		
	*n = 299*	*n = 166*	*n = 133*	Crude OR	95% CI	*p-value*
Social and Economic						
*Gender*						
Male	154 (51.5)	90 (54.2)	64 (48.1)	Ref.	Ref.	
Female	145 (48.5)	76 (45.8)	69 (51.9)	0.78	(0.50 to 1.24)	0.295
*Age, mean ± SD*	65.79 ± 13.7	68.96 ± 12.8	61.83 ± 13.9	1.49	(1.24 to 1.78)	**< 0.001**
* Immigration Status*						
Born in Spain	251 (83.9)	156 (94.0)	95 (71.4)	Ref.	Ref.	
Immigrated to Spain	48 (16.1)	10 (6.0)	38 (28.6)	0.160	(0.08 to 0.34)	**< 0.001**
*Household Income*						
1st Tertile (lowest income)	100 (33.44)	52 (31.33)	48 (36.09)	Ref.	Ref.	
2nd Tertile	100 (33.44)	62 (37.35)	38 (28.57)	1.51	(0.86 to 2.65)	0.154
3er Tertile (highest income)	99 (33.11)	52 (31.33)	47 (35.34)	1.02	(0.59 to 1.78)	0.941
* Living Status*						
Living alone	63 (21.1)	28 (16.9)	35 (26.3)	Ref.	Ref.	
Living with someone	236 (78.9)	138 (83.1)	98 (73.7)	1.69	(1.01 to 3.08)	**0.048**
* Education*						
Primary school or lower	176 (58.9)	98 (59.0)	78 (58.7)	Ref.	Ref.	
Secondary school	88 (29.4)	51 (30.7)	37 (27.8)	1.10	(0.65 to 1.84)	0.726
University or higher	35 (11.7)	17 (10.2)	18 (13.5)	0.75	(0.36 to 1.55)	0.441
Healthcare Team and System-Related						
* Frequency of Follow-up Care*						
Monthly or more frequently	152 (50.8)	81 (48.8)	71 (53.4)	Ref.	Ref.	
Quarterly	117 (39.1)	64 (38.6)	53 (39.8)	1.06	(0.65 to 1.72)	0.818
Biannually or less frequently	30 (10.0)	21 (12.7)	9 (6.8)	2.05	(0.88 to 4.75)	0.096
*Number of Pharmacies Used for Refills, mean ± SD*	1.64 ± 1.0	1.35 ± 0.8	2.00 ± 1.2	0.51	(0.40 to 0.66)	**< 0.001**
* Patient-provider Communication*						
Not having good communication	24 (8.0)	9 (5.4)	15 (11.3)	Ref.	Ref.	
Having good communication	275 (92.0)	157 (94.6)	118 (88.7)	2.22	(1.03 to 6.11)	0.070
* Quality of Healthcare Delivery*						
Not perceiving good quality of care	13 (4.3)	5 (3.0)	8 (6.0)	Ref.	Ref.	
Perceiving good quality of care	286 (95.7)	161 (97.0)	125 (94.0)	2.06	(0.66 to 6.45)	0.214
* Treatment Information Received*						
Not receiving complete information	161 (53.8)	60 (36.1)	101 (75.9)	Ref.	Ref.	
Receiving complete information	138 (46.2)	106 (63.9)	32 (24.1)	5.58	(3.35 to 9.27)	**< 0.001**
Condition-Related						
*Number of Chronic Conditions, mean ± SD*	2.90 ± 1.5	3.08 ± 1.6	2.68 ± 1.5	1.19	(1.02 to 1.39)	**0.027**
*Adjusted Morbidity Group*						
AMG1	62 (20.7)	29 (17.5)	33 (24.8)	Ref.	Ref.	
AMG2	163 (54.5)	89 (53.6)	74 (55.6)	1.37	(0.76 to 2.46)	0.294
AMG3	74 (24.8)	48 (28.9)	26 (19.5)	2.10	(1.05 to 4.19)	**0.035**
* Alcohol Consumption*						
Daily drinker	59 (19.7)	30 (18.1)	29 (21.8)	Ref.	Ref.	
Occasional drinker	101 (33.8)	51 (30.7)	50 (37.6)	0.99	(0.52 to 1.87)	0.966
Non-drinker	139 (46.5)	85 (51.2)	54 (40.6)	1.52	(0.82 to 2.81)	0.824
* Tobacco Smoking*						
Smoker	49 (16.4)	30 (18.1)	19 (14.3)	Ref.	Ref.	
Ex-smoker	98 (32.8)	56 (33.7)	42 (31.6)	0.84	(0.42 to 1.70)	0.636
Non-smoker	152 (50.8)	80 (48.2)	72 (54.1)	0.70	(0.37 to 1.36)	0.294
* Physical Activity*						
Non-active	128 (42.8)	64 (38.6)	64 (48.1)	Ref.	Ref.	
Active	171 (57.2)	102 (61.4)	69 (51.9)	0.68	(0.43 to 1.07)	0.097

*Abbreviations*: *CI*, confidence interval; *OR*, odds ratio; *SD*, standard deviation; *Ref*., reference

Bold values are statistically significant at *p*-values ≤0.05

### Adherence to treatment

The proportion of adherent patients to treatment according to the Morisky-Green-Levine questionnaire was 55.5%. Medium and low adherence rates were 39.8% and 4.7% respectively and comprised the “poor-adherent group” (44.5%). The likely causes for non-adherence among the poor-adherent group were occasionally forgetting to take medications (79.0%), being careless at times about taking medications (29.3%), discontinuing medications when feeling better (21.1%), and discontinuing medications when feeling worse (24.1%) (Additional file 1: Table S2).

### Factors associated with adherence

**Fig. 1:**
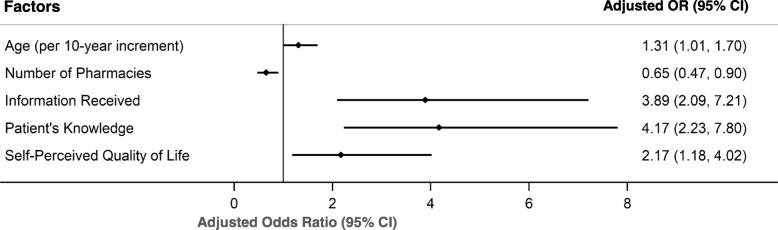
Multivariate Logistic Regression Analysis. The forest plot displays the Adjusted Odds Ratio (OR) and 95% Confidence Interval (CI) for factors associated with medication adherence — older age, lower number of pharmacies used for medication refills, having received complete treatment information, having adequate knowledge about medication regimen, and self-perception of a good quality of life — among patients with chronic conditions in Primary Care. The model was further adjusted for immigration status, living status, number of chronic conditions, adjusted morbidity group, interference of therapy on daily life activities, and use of medication dosing reminders. *Abbreviations*: *OR*, odds ratio; *CI*, confidence interval

Bivariate logistic analyses revealed several factors associated with medication adherence in all the WHO dimensions (Table 1 and Table 2). Variables significantly associated with adherence (*p*-value ≤ 0.05) were included in the multivariable logistic regression analyses. After multivariable adjustment, five factors were independently associated with adherence (Table 3). Participants who were older (adjusted odds ratio 1.31 per 10-year increment, 95% confidence interval [CI] 1.01–1.70), refilled prescriptions in lower number of pharmacies (0.65, 95% CI 0.47–0.90), received complete information about treatment (3.89, 95% CI 2.09–7.21), had adequate knowledge about medication regimen (4.17, 95% CI 2.23–7.80), and self-perceived of having good quality of life (2.17, 95% CI 1.18–4.02) were more likely to adhere to treatment schedule regimens (Fig. 1).

**Table 2:** Therapy- and Patient-Related Factors of the Participants of the Study according to their Self-Reported Measure of Medication Adherence, *n* = 299

Factors	Total n (%)	Adherent n (%)	Poor Adherent n (%)	Bivariate Analyses		
	*n = 299*	*n = 166*	*n = 133*	Crude OR	95% CI	*p-value*
Therapy-Related						
*Number of prescriptions, mean ± SD*	3.90 ± 2.2	3.94 ± 2.2	3.85 ± 2.3	1.02	(0.92 to 1.13)	0.725
*Number of pills, mean ± SD*	4.36 ± 2.9	4.26 ± 2.9	4.50 ± 3.0	0.97	(0.90 to 1.05)	0.482
*Medication through injections*						
Using injections	33 (11.0)	18 (10.8)	15 (11.3)	Ref.	Ref.	
Not using injections	266 (89.0)	148 (89.2)	118 (88.7)	1.05	(0.51 to 2.16)	0.905
*Therapy through inhalers*						
Using inhalers	28 (9.4)	14 (8.4)	14 (10.5)	Ref.	Ref.	
Not using inhalers	271 (90.6)	152 (91.6)	119 (89.5)	1.28	(0.59 to 2.78)	0.538
*Interfering with Activities of Daily Living*						
Interfering	27 (9.03)	10 (6.0)	17 (12.8)	Ref.	Ref.	
Not-interfering	272 (91.0)	156 (94.0)	116 (87.2)	2.29	(1.01 to 5.18)	**0.047**
Patient-Related						
*Functional Independency of Daily Living Activities*						
Independent	234 (78.3)	127 (76.5)	107 (80.5)	Ref.	Ref.	
Slightly dependent	49 (16.4)	29 (17.5)	20 (15.0)	1.22	(0.65 to 2.28)	0.530
Moderately dependent	16 (5.4)	10 (6.0)	6 (4.5)	1.40	(049 to 3.99)	0.524
Severely dependent	0 (0)	0 (0)	0 (0)	–	–	–
Totally dependent	0 (0)	0 (0)	0 (0)	–	–	–
*Medication Dosing Reminders*						
Not using any reminder	85 (28.4)	27 (16.3)	58 (43.6)	Ref.	Ref.	
Use of alarms/phones/pillboxes	80 (26.8)	50 (30.1)	30 (22.6)	3.58	(1.88 to 6.81)	**< 0.001**
Association of medication with daily routines	134 (44.8)	89 (53.6)	45 (33.8)	4.25	(2.38 to 7.59)	**< 0.001**
*Patient’s Knowledge about Medication Regimen*						
Not having an adequate knowledge	173 (57.9)	75 (45.2)	98 (73.7)	Ref.	Ref.	
Having an adequate knowledge	126 (42.1)	91 (54.8)	35 (26.3)	3.40	(2.08 to 5.56)	**< 0.001**
*Perceived Overmedication in the Treatment*						
Perception of overmedication	26 (8.7)	12 (7.2)	14 (10.5)	Ref.	Ref.	
Not-perception of overmedication	273 (91.3)	154 (92.8)	119 (89.5)	1.51	(0.67 to 3.39)	0.317
*Self-Perceived Quality of Life*						
Not having good quality of life	153 (51.2)	76 (45.8)	77 (57.9)	Ref.	Ref.	
Having good quality of life	146 (48.8)	90 (54.2)	56 (42.1)	1.63	(1.03 to 2.58)	**0.038**

*Abbreviations: CI*, confidence interval; *OR*, odds ratio; *SD*, standard deviation; *Ref*., reference

Bold values are statistically significant at *p*-values ≤0.05

**Table 3:** Multivariable Logistic Regression Models between Factors in the WHO’s Domains and Medication Adherence as Measured by the Four-Item Morisky-Green-Levine Self-Reported Questionnaire, n = 299

	Multivariate Logistic Regression		
Factors	Adjusted OR	95% CI	*p-value*
Social and Economic			
*Age* (*per 10-year increment)*	1.31	(1.01–1.70)	**0.039**
*Immigration Status*			
Born in Spain	Ref.	Ref.	
Immigrated to Spain	0.64	(0.25 to 1.65)	0.352
*Living Status*			
Living alone	Ref.	Ref.	
Living with someone	1.81	(0.89 to 3.68)	0.099
Healthcare Team and System-Related			
*Number of Pharmacies Used for Refills*	0.65	(0.47 to 0.90)	**0.008**
*Treatment Information Received*			
Not receiving complete information	Ref.	Ref.	
Receiving complete information	3.89	(2.09 to 7.21)	**< 0.001**
Condition-Related Factors			
*Number of Chronic Conditions*	1.31	(0.99 to 1.73)	0.061
*Adjusted Morbidity Group*			
AMG1	Ref.	Ref.	
AMG2	0.68	(0.28 to 1.69)	0.410
AMG3	0.84	(0.28 to 2.79)	0.836
Therapy-Related			
*Interfering with Activities of Daily Living*			
Interfering	Ref.	Ref.	
Not-interfering	1.52	(0.53 to 4.34)	0.432
Patient-Related			
*Medication Dosing Reminders*			
Not using any reminder	Ref.	Ref.	
Use of alarms/phones/pillboxes	1.56	(0.69 to 3.52)	0.281
Association of medication with daily routines	1.55	(0.74 to 3.28)	0.244
*Patient’s Knowledge about Medication Regimen*			
Not having an adequate knowledge	Ref.	Ref.	
Having an adequate knowledge	4.17	(2.23 to 7.80)	**< 0.001**
*Self-Perceived Quality of Life*			
Not having good quality of life	Ref.	Ref.	
Having good quality of life	2.17	(1.18 to 4.02)	**0.013**

*Abbreviations: CI*, confidence interval; *OR*, odds ratio; *Ref*., reference

Bold values are statistically significant at *p*-values ≤0.05

### Sensitivity analyses

To test the robustness of our results and the validity and reliability of the methods used for assessing adherence, we conducted multiple linear regression analysis using patient’s overall medication score (0–4 score) as continuous dependent variable. Age, the number of pharmacies used to refill prescriptions, treatment information received, and knowledge about treatment were consistently associated with medication adherence (Table 4).

**Table 4:** Multiple Linear Regression Analysis of Factors of Adherence using the Morisky-Green-Levine’s Scale Score, *n* = 299

	Multivariate Linear Regression Model			
Factors	Correlation Coeficient	SE	95% CI	*p-value*
Social and Economic				
*Age* (*per 10-year increment)*	−0.15	0.04	(−0.23 to −0.07)	**0.001**
*Immigration Status*				
Born in Spain	Ref.		Ref.	
Immigrated to Spain	0.27	0.15	(−0.02 to 0.56)	0.070
*Living Status*				
Living alone	Ref.		Ref.	
Living with someone	− 0.18	0.11	(− 0.40 to 0.04)	0.107
Healthcare Team and System-Related				
*Number of Pharmacies Used for Refills*	0.14	0.05	(0.04 to 0.24)	**0.005**
^*1*^*Treatment Information Received*	−0.20	0.05	(− 0.30 to − 0.10)	**< 0.001**
Condition-Related Factors				
*Number of Chronic Conditions*	−0.07	0.04	(−0.16 to 0.01)	0.077
*Adjusted Morbidity Group*				
AMG1	Ref.		Ref.	
AMG2	0.11	0.14	(−0.16 to 0.39)	0.427
AMG3	0.15	0.18	(−0.21 to 0.51)	0.412
Therapy-Related				
*Interfering with Activities of Daily Living*				
Interfering	Ref.		Ref.	
Not-interfering	0.06	0.16	(−0.27 to 0.38)	0.736
Patient-Related				
*Medication Dosing Reminders*				
Not using any reminder	Ref.		Ref.	
Use of alarms/phones/pillboxes	0.02	0.14	(−0.25 to 0.29)	0.878
Association of medication with daily routines	−0.17	0.13	(−0.42 to 0.09)	0.200
^*1*^*Knowledge about Medication Regimen*	−0.17	0.05	(−0.26 to − 0.08)	**< 0.001**
^*1*^*Self-Perceived Quality of Life*	−0.05	0.04	(−0.14 to 0.04)	0.264

*Abbreviations: SE*, standard error; *CI*, confidence interval; *Ref*., reference

Bold values are statistically significant at *p*-values ≤0.05

^1^Introduced as Likert scale score (1–5)

## Discussion

To our knowledge, this is the first attempt to determine factors associated with adherence under the WHO multidimensional framework in patients with chronic conditions in primary care settings in Spain. The results showed that slightly more than half of the subjects of the cohort remained adherent to long-term therapies for chronic conditions, which points to substantial room for improvement at the primary-care level. Forgetfulness was the main likely cause of non-adherence among the poor adherence group. After adjustment for several variables, we found age, the number of pharmacies used for medication refills, the treatment information received, patients’ knowledge about medication regimen, and self-perceived quality of life as independent factors of adherence.

The adherence rate found in our study was consistent with the WHO report that states “in developed countries, adherence among patients suffering chronic diseases averages only 50%” [9]. Compared with previous international studies, the adherence rate assessed in this study was similar to the 53% found in Chinese primary-care centers [31], the 48% reported in uninsured American patients who attended community health centers [32], but slightly higher than the 39% observed in Italian outpatient adults [24]. At the national level, our findings are consistent with prior research in Spain performed in chronic patients [33], but significantly different in terms of the adherence rate of 18% reported in tertiary-care settings [23].

Patient’s knowledge about medication regimen provided the strongest predictor of adherence. A large proportion of participants found it difficult to explain the amount, number, and frequency of doses associated with their medications, negatively affecting their adherence. For patients with chronic conditions, understanding of their own diseases and the complex regimens may represent a challenge [34]. For example, Friis et al. [35] found that individuals with long-term diseases had more difficulties in comprehending provider health information. Similarly, Fredericksen et al. [36] and Kvarnström et al. [37] reported frequent misconceptions and lack of understanding of the purpose of medications among the chronically ill.

Our analyses also proved that treatment information was an important predictor of adherence. Clear, unbiased, and proper information improves patients’ understanding of their treatment, increases awareness of benefits and risks of medication, and sets realistic expectations, which improves adherence [38]. Nonetheless, patients frequently receive little information about treatment during clinical consultations [39] and have needs and concerns that are not addressed [40].

General practitioners (GPs), responsible for much of the prescribing medication and counseling for chronic conditions [18–20], have reported time pressure as a frequent barrier for informing and educating patients in primary care settings [38]. Moreover, physicians have acquired strategies to manage consultation times by interrupting patients before giving them the opportunity to explain their concerns completely [41]. Such circumstances may lead patients to experience greater frustration, and to wish that their provider had more time to spend talking to them addressing their concerns [42].

To improve patients’ education and to tackle the non-adherence concern, GPs have enlisted more cooperation from other allied health professionals such as nurses and pharmacists [37]. They can play an important role in patients’ education and counseling. Nurses can educate patients by providing information on diseases and patients’ diagnosis [43, 44]. Additionally, they can promote self-management of chronic conditions and support medication adherence. Similarly, pharmacists may enrich patient’s education by providing information regarding medication such as proper use of drugs, potential side effects and interactions, dosing schedules, and healthy lifestyles [43, 44]. Likewise, pharmacists have enhanced medication adherence by using motivational interviewing skills, reviewing patients’ regimens, supervising treatment efficacy and security, and discussing the management of missed doses. [45]. These factors suggest the importance of strengthening collaboration between GPs, nurses, and pharmacists to improve patients’ care. Patients also believe that interprofessional collaborations are needed to provide the best care possible [46].

We found that, as the number of pharmacies used for refilling prescriptions increased, treatment adherence decreased. This is consistent with previous studies that have found patients who made more visits to more pharmacies and those with less refill consolidation were substantially less adherent to their therapies [47, 48]. This finding underlies the importance of the role of pharmacist in the context of medication adherence.

The use of a single pharmacy allows patients to have a long-term relationship with pharmacists that fosters pharmacist-patient communication and counseling. Use of only one pharmacy to refill prescriptions also facilitates the pharmacist’s ability to track patients’ medication, improves patients’ follow up, and establishes a consistent medication record. Having a pharmacy-based computer system connecting all pharmacies may be one possible approach; however, it may lead patient to confusion in managing medications and hinder communication between patient, physicians, nurses, and pharmacists. Moreover, pharmacists have reported a lack of confidence in having a complete idea of medication lists when patients use multiple pharmacies and may be less likely to optimize drug utilization and safety [49]. Use of a single pharmacy, commonly called a “pharmacy home”, has been proposed as a helpful way to foster patient-pharmacist communication and maintain a better control of medication [47, 48, 50]. However, a pharmacy home may be impractical for many patients and increase out of pocket costs [51]. As such, integrating pharmacists into primary care, as previously discussed, may represent the most evidence-based and feasible approach. Such an example occurs in North Carolina, where clinical pharmacist practitioners are integrated into primary care in team-based models of care having positive impact on clinical and cost outcomes [52, 53].

Self-perception of a good quality of life and older age were also associated with adherence. Nonetheless, a lack of consensus exists about their precise effect. While some studies corroborate our findings suggesting a relationship between quality of life and adherence attributed to the influence of some psycho-social characteristics related to the ability of manage chronic diseases [54], others have not found such association [55]. Similarly, the effect of age has been inconsistent across adherence studies. An increase in age is generally associated with a greater adherence as younger people may perceive less severity of disease. This association continues until the onset of some aging processes, such as cognitive impairments, which usually occurs around the 70 years of age, in which adherence starts to decline [56].

Our results should be interpreted in light of several limitations. Self-reported questionnaires use for measuring adherence may be susceptible to recall bias and may underestimate the true extent of non-adherence [57]. Nonetheless, the Morisky-Green-Levine questionnaire has yielded fair psychometric properties (sensitivity = 0.81, specificity = 0.44), and provided a useful tool to evaluate medication adherence in numerous chronic disease studies. Furthermore, patients may want to please their healthcare providers with their responses and may incur in social desirability bias. To minimize this problem, the interviewer was not affiliated with the study sites and had no contact with participants prior to the interview. Another limitation reflects the nature of the study itself. The cross-sectional design may limit evaluation of cause-effect relationships. Longitudinal studies should explore the temporal validity of the associations found here. Lastly, the reference proportion of participants not adhering to prescribed medications proposed to calculate the sample size of the study differs from the final findings, which may have somewhat underpowered our results. Nonetheless, study’s strengths rest in the assessment of overall adherence in patients with chronic diseases rather than adherence to one single condition or particular drug therapy. We have considered a number of common chronic conditions and evaluated several factors using the WHO conceptual framework. Furthermore, since multimorbidity has become the rule rather the exception in primary care settings [20], our research provides a more realistic and accurate assessment of the non-adherence problem.

## Conclusions

Adherence to long-term treatments for chronic conditions remains a challenging issue in primary care. A low proportion of patients followed the recommendations from healthcare providers which underlines the need of reinforcing medication adherence in primary care. Our results should help to design new interventions aimed to enhance adherence. Considerable attention should be given to the multidimensional factors potentially amenable to intervention found in this study such as patient’s knowledge and information. Health professionals should emphasize on meeting patients’ information needs and reinforcing their education on treatment and diseases. Our results also provide firm evidence of the positive impact of pharmacists on patients’ adherence when having a consolidated relationship. Due to current and future challenges in primary care, future research is needed to evaluate the extent of integrating pharmacists into new team-based models of primary care.

## Additional file

Additional file 1: **Table S1.** Proportion of Study Participants with Chronic Conditions. **Table S2.** Level of Adherence and Likely Causes of Non-Adherence according the Morisky-Green-Levine Questionnaire. (DOCX 16 kb)

## Data Availability

Data will be available upon reasonable request from the corresponding author.

## Abbreviations

AMG: Adjusted morbidity group
CI: Confidence interval
GPs: General practitioners
OR: Odds ratio
Ref: Reference
SD: Standard deviation
SE: Standard error
SNHS: Spanish National Health System
WHO: World Health Organization
