# Supplementary material for: Adherence to treatment and related factors among patients with chronic conditions in primary care: a cross-sectional study
Source: BMC Fam Pract. 2019 Sep 14;20:132. doi: 10.1186/s12875-019-1019-3 (PMC6744672; doi:10.1186/s12875-019-1019-3)
Supplement: Supplementary file 1 — Table S1. Proportion of Study Participants with Chronic Conditions. Table S2. Level of Adherence and Likely Causes of Non-Adherence according the Morisky-Green-Levine Questionnaire. (DOCX 16 kb) [file 12875_2019_1019_MOESM1_ESM.docx]

**Supplementary Material**

| **Table s1:** Proportion of Study Participants with Chronic Conditions | | |
| --- | --- | --- |
| **Chronic Conditions Included in the Study Defined by the ICD-10 diagnoses code** | **Total**  **n (%)** |  |
| **Circulatory System Diseases**  Hypertensive disease (I10-I15), ischemic heart disease (I20-I25), heart failure (I50), and cerebrovascular disease (stroke) (I60-I69). | 213 (71.2%) |  |
| **Respiratory System Diseases**  Chronic obstructive pulmonary disease (J41-J44) and asthma currently treated (J45-J46). | 53 (17.7%) |  |
| **Mental and Nervous System Diseases**  Minor depressive disorder (F32.0, F33.0), Alzheimer’s disease (G30), Parkinson’s disease (G20-G21), multiple sclerosis (G35), and epilepsy currently treated (G40-G41). | 113 (37.8%) |  |
| **Endocrine and Metabolic Disorders**  Thyroid disorders (E00-E03), diabetes mellitus (E10-E14), and hyperlipidemia (E78). | 159 (53.2%) |  |
| **Genitourinary System Diseases**  Chronic kidney disease (N18) and benign prostatic hyperplasia (N40). | 31 (10.4%) |  |
| **Musculoskeletal System Diseases**  Rheumatoid arthritis (M05-M06), arthrosis (M10-M11), gout (M15-M19), and painful condition (≥4 analgesic prescriptions in last 12 months). | 60 (20.1%) |  |
| **Sense Organs Diseases and Infections**  HIV/AIDS (B20-B24), viral hepatitis (B15-B19), and glaucoma (H40-H42). | 18 (6.0%) |  |

| **Table s2:** Level of Adherence and Likely Causes of Non-Adherence according the Morisky-Green-Levine Questionnaire | |
| --- | --- |
| **Level of adherence** | *(n=299)* |
| High level (0 score) | 166 (55.5%) |
| Medium level (1-2 score) | 119 (39.8%) |
| Low level (≥3 score) | 14 (4.7%) |
| **Likely causes of non-adherence**  **among the poor adherence group** | *(n=133)* |
| Forgetting to take medications | 105 (79.0%) |
| Careless at times about taking medications | 39 (29.3%) |
| Feeling better | 28 (21.1%) |
| Feeling worse | 32 (24.1%) |
